# The short- and long-term readmission of four major categories of digestive system cancers: does obesity or metabolic disorder matter?

**DOI:** 10.3389/fendo.2023.1214651

**Published:** 2023-10-30

**Authors:** Yan Li, Xiaoqin Wu, Yongfeng Song, Peipei Wang, Bofei Zhang, Bingzhou Guo, Ziwei Liu, Yafei Wu, Shanshan Shao, Yiping Cheng, Honglin Guo, Xiude Fan, Jiajun Zhao

**Affiliations:** ^1^ Department of Endocrinology, Shandong Provincial Hospital, Shandong University, Jinan, China; ^2^ Shandong Clinical Research Center of Diabetes and Metabolic Diseases, Jinan, Shandong, China; ^3^ Shandong Institute of Endocrine and Metabolic Diseases, Jinan, Shandong, China; ^4^ Shandong Engineering Laboratory of Prevention and Control for Endocrine and Metabolic Diseases, Jinan, Shandong, China; ^5^ Shandong Engineering Research Center of Stem Cell and Gene Therapy for Endocrine and Metabolic Diseases, Jinan, Shandong, China; ^6^ Department of Inflammation and Immunity, Cleveland Clinic, Cleveland, OH, United States; ^7^ Department of Endocrinology, Shandong Provincial Hospital Affiliated to Shandong First Medical University, Jinan, China; ^8^ College of Artificial Intelligence and Big Data for Medical Sciences, Shandong First Medical University and Shandong Academy of Medical Sciences, Jinan, China

**Keywords:** obesity, metabolic, phenotype, digestive system, neoplasm

## Abstract

**Purpose:**

Patients with digestive system cancers (DSCs) are at a high risk for hospitalizations; however, the risk factors for readmission remain unknown. Here, we established a retrospective cohort study to assess the association between metabolic obesity phenotypes and readmission risks of DSC.

**Experimental design:**

A total of 142,753 and 74,566 patients at index hospitalization were ultimately selected from the Nationwide Readmissions Database (NRD) 2018 to establish the 30-day and 180-day readmission cohorts, respectively. The study population was classified into four groups: metabolically healthy non-obese (MHNO), metabolically healthy obese (MHO), metabolically unhealthy non-obese (MUNO), and metabolically unhealthy obese (MUO). Multivariate Cox regression analysis was used to estimate the effect of metabolic obesity phenotypes on DSC readmission.

**Results:**

The MUNO phenotype had 1.147-fold (95% CI: 1.066, 1.235; *p* < 0.001) increased 180-day readmission risks in patients with neoplasm of the upper digestive tract. The MUNO phenotype had 1.073-fold (95% CI: 1.027, 1.121; *p* = 0.002) increased 30-day readmission risks and 1.067-fold (95% CI: 1.021, 1.115; *p* = 0.004) increased 180-day readmission risks in patients with neoplasm of the lower digestive tract. The MUNO and MUO phenotypes were independent risk factors of readmission in patients with liver or pancreatic neoplasm. Metabolic obesity status was independently associated with a high risk of severe and unplanned hospitalization within 30 days or 180 days.

**Conclusion:**

Both obesity and metabolic abnormalities are associated with a high risk for the poor prognosis of DSC patients. The effect of metabolic categories on the short- or long-term readmission of liver or pancreas cancers may be stronger than that of obesity.

## 1 Introduction

The fast-growing incidence of digestive system cancers (DSCs) worldwide is juxtaposed with a notable increase in mortality. A status report on the global burden of cancer worldwide using the Global Cancer Statistics 2018 (1) estimated that major DSCs (esophagus cancer, stomach cancer, colorectal cancer, liver cancer, and pancreas cancer) come in at 29.6% of newly diagnosed cancers and 39.0% of all cancer deaths. Therefore, DSCs have brought a substantial and heavy global health burden (2, 3). Of note, cancer patients are at a high risk of readmission due to the inherent complexity of their medical, comorbid, and psychosocial conditions (4). Furthermore, unplanned readmissions may cost the Centers for Medicare & Medicaid Services (CMS) an estimated >$17 billion annually (5, 6). While some inevitable readmissions occur as a result of the natural course of the disease, a proportion can be intervened in advance by identifying factors associated with hospital readmission for these high-risk patients early so that effective prevention and intervention strategies can be established to optimize short-term and long-term clinical outcomes while reducing costs (7, 8).

Obesity increases the hospital readmission risk of various diseases, such as acute myocardial infarction (9), end-stage renal disease (10), and colorectal cancer after surgery (11). However, recent studies now report that the obesity paradox exists and may even be extended to postoperative outcomes following robotic-assisted surgery for rectal cancer, that is, obesity did not impact the readmission of such patients (12). So, what accounts for this? Of note, not all obesity phenotypes are created equally, and obesity often naturally coexists with insulin resistance, dyslipidemia, and hypertension (13). However, it also cannot be ignored that obesity is not always associated with metabolic abnormalities. A metabolically healthy phenotype, known as metabolically healthy obesity (MHO), may occur in approximately 25%–30% of the obese population (14–17). Kabat GC et al. have yielded arrestive results that metabolically unhealthy normal weight (MUNW) phenotypes were associated with an increased risk of colorectal cancer, while other phenotypes showed no association (18). Therefore, the combination of obesity phenotype and different metabolic characteristics seems to be more accurate than obesity alone in predicting the readmission risk of diseases.

However, no study has yet systematically examined the association between metabolically defined obesity type and the short- or long-term readmission risk of DSC (neoplasm of the esophagus, stomach, duodenum, small intestine, large intestine, rectum, anal canal, anus, liver, and pancreas). The purpose of this study is to understand this important knowledge gap in two readmission cohorts by combining obesity and metabolic health status into a composite variable to assess the individual and joint effects of the two exposures and provide a reference for clinical prevention and readmission reduction programs.

## 2 Methods

### 2.1 Data sources and extraction

We utilized the Nationwide Readmissions Database (NRD) 2018 to study 30-day readmission and 180-day readmission among patients with four major categories of DSCs, namely, neoplasm of the upper digestive tract, lower digestive tract, liver, and pancreas. The NRD, as a part of the Healthcare Cost and Utilization Project (HCUP), contains all-payer hospital inpatient stays data from 28 geographically dispersed states, representing 51% of the total U.S. population and 49% of all U.S. hospitalizations to generate national estimates of readmissions to hospital (19). Using a reliable and verified linkage number, patients are identified to track across the hospitals.

Using the corresponding International Classification of Diseases-10th Revision-Clinical Modification (ICD-10-CM) diagnostic and procedure codes, a total of 12,928,231 patients who were admitted to the hospital in 2018 were identified in the NRD database. There are no ethical aspects to this study.

### 2.2 Study population

**Figure 1:**
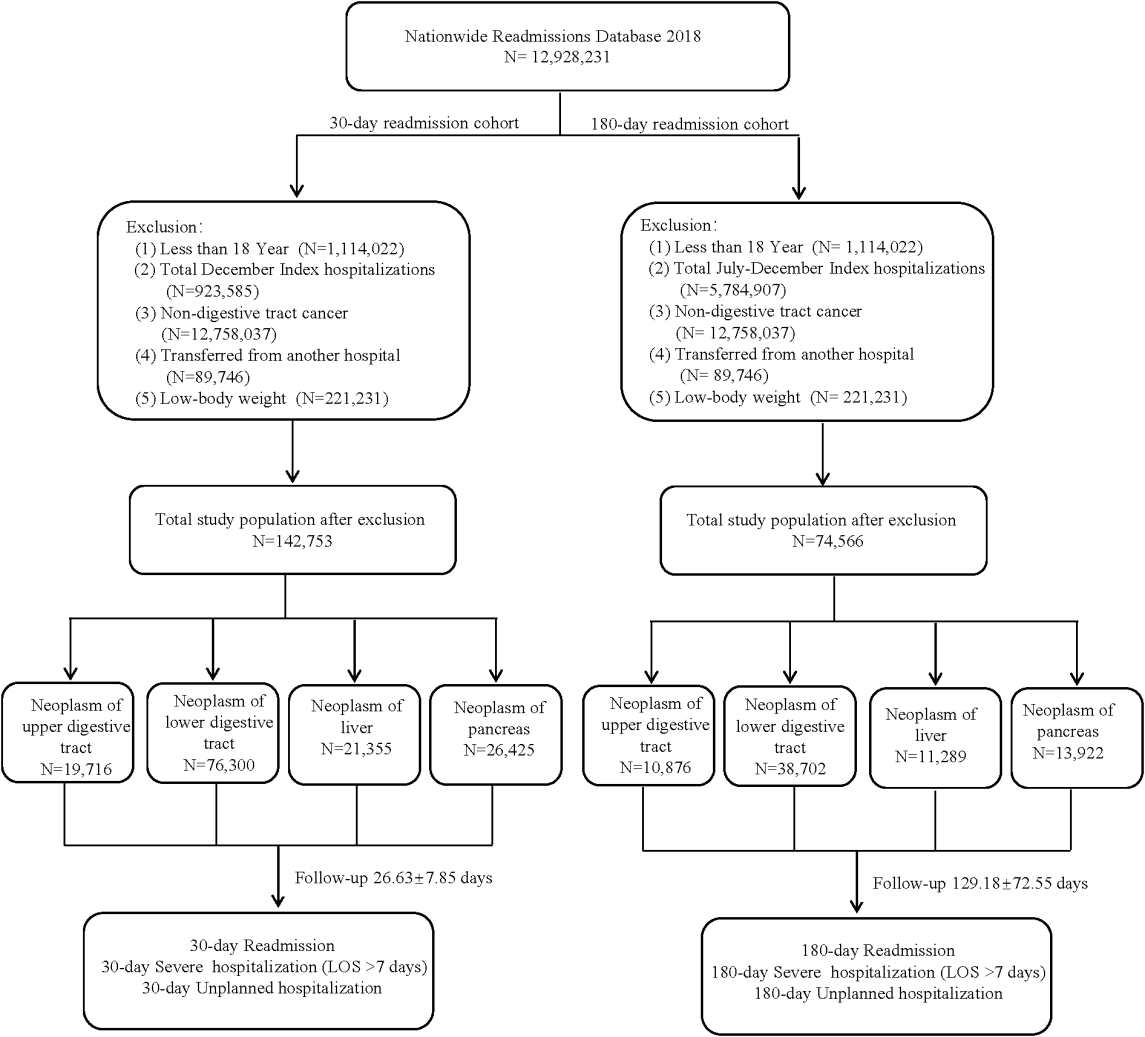
Flowchart of the study. LOS, length of stay.

For the 30-day readmission cohort, admissions in December (*N* = 923,585) were excluded, and for the estimation of the 180-day readmission, admission in the months of July, August, September, October, November, and December (*N* = 5,784,907) were excluded, as admission in the next year cannot be tracked in the NRD. We also excluded patients with 1) age less than 18 at the time of index hospitalization, 2) non-digestive system cancer, 3) transfer from another hospital, and 4) low body weight. In total, 142,753 and 74,566 participants with a primary or secondary discharge diagnosis of digestive system cancer at the time of index hospitalization were ultimately selected for inclusion in the short- or long-term readmission risk analysis, respectively. The details of the inclusion and exclusion criteria are described in **Figure 1**. Index hospitalization was defined as the whole hospital stay during which the index stay occurred. If a patient had two or more hospitalizations, the first hospitalization was considered as the index hospitalization.

### 2.3 Exposure assessment

Obesity was defined as body mass index (BMI) ≥25 kg/m (2). Metabolically unhealthy status was defined as having at least one of the following three components of metabolic syndrome (20) except for waist circumference collinear with BMI: 1) hyperglycemia; 2) hyperlipidemia: high serum triglyceride (TG) levels or high high-density lipoprotein (HDL)-cholesterol levels, etc.; and 3) hypertension. Metabolic health status was defined as having none of the above three components of metabolic syndrome. These diagnoses identified by the ICD-10-CM codes are listed in **Supplementary Table 1**. Furthermore, we used a combination of obesity and metabolic status to classify individuals into metabolic obesity phenotypes as follows: 1) MHNO: metabolically healthy non-obese, metabolic health status with BMI <25 kg/m (2); 2) MHO: metabolically healthy obese, metabolic health status with BMI ≥25 kg/m (2); 3) MUNO: metabolically unhealthy non-obese, metabolically unhealthy status with BMI <25 kg/m (2); and 4) MUO: metabolically unhealthy obese, metabolically unhealthy status with BMI ≥25 kg/m (2).

### 2.4 Patient characteristics

DSC in our study refers to all types of neoplasm combined including neoplasm of the esophagus, stomach, duodenum, small intestine, large intestine, rectum, anal canal, anus, liver, and pancreas. Neoplasm of the upper digestive tract was defined as neoplasm combined including neoplasm of the esophagus, stomach, and duodenum. Neoplasm of the lower digestive tract included the small intestine, large intestine, rectum, anal canal, and anus. Furthermore, patient demographics, including age, sex, income quartile based on household income of the patient’s zip code, patient location, length of stay (LOS), primary expected payment source (Medicare/Medicaid, private insurance, self-pay, and other insurance types), and common comorbidities to calculate Charlson’s comorbidity, were also obtained from the NRD database according to the corresponding code.

### 2.5 Outcomes

Our primary outcomes of interest were short- (30-day) or long-term (180-day) readmission risk of DSC after discharge from index hospitalization. Secondary outcomes of interest included the following: 1) severe DSC-related readmission (LOS >7 days) and 2) unplanned hospitalization.

In addition, we counted the top 4 main reasons for readmission of patients with different metabolic obesity phenotypes of DSC based on primary diagnosis codes.

### 2.6 In-depth subgroup analysis

To better elucidate the effect of obesity, hyperglycemia, hyperlipidemia, and hypertension on the short- or long-term readmission risk of DSC, the study population was divided into 10 groups and 8 groups twice for comparisons. The first grouping included those with 1) non-obese and no metabolic abnormality, 2) non-obese and simple hyperglycemia, 3) non-obese and simple hypertension, 4) non-obese and simple hyperlipidemia, 5) non-obese and multiple metabolic abnormalities, 6) obese and no metabolic abnormality, 7) obese and simple hyperglycemia, 8) obese and simple hypertension, 9) obese and simple hyperlipidemia, and 10) obese and multiple metabolic abnormalities. The secondary grouping included 1) non-obese and no metabolic abnormality, 2) non-obese and one metabolic syndrome component, 3) non-obese and two metabolic syndrome components, 4) non-obese and three metabolic syndrome components, 5) obese and no metabolic abnormality, 6) obese and one metabolic syndrome component, 7) obese and two metabolic syndrome components, and 8) obese and three metabolic syndrome components.

### 2.7 Statistical analysis

We used descriptive statistics to compare patient demographic characteristics and the prevalence of DSC across four groups of metabolic obesity phenotypes. Continuous variables were expressed as median ± standard deviation and categorical variables as frequency counts and percentages. The chi-square test was used to compare categorical variables, while variance testing was used to compare continuous variables. The multivariable Cox regression model was performed to identify higher risk subgroups of the 30-day or 180-day readmission of DSC and secondary outcomes of interest, using MHNO, a low-risk group, as the reference. To report adjusted hazard ratios (HRs) for DSC, age, sex, primary expected payment source, patient location, Deyo–Charlson comorbidity index, LOS at index hospitalization, and severe hospitalization at index hospitalization were selected as covariates. Kaplan–Meier curves for the probability of outcomes were obtained for the four phenotypes of different DSCs. R Studio (2022.12.0 + 353), R (4.1.1), and SPSS 25.0 software (SPSS Inc., Chicago, IL, USA) were used to conduct all statistical analyses in our study. A two-sided *p*-value <0.05 was considered significant.

## 3 Results

### 3.1 Patient characteristics based on metabolic obesity phenotype at index hospitalization

In **Table 1**, we described the sociodemographic and common comorbidity characteristics and prevalence of DSC in patients with different metabolic obesity phenotypes. Patients in the MHO categories had the lowest age level and male proportions in the 30-day and 180-day readmission cohorts. Compared with patients with MHNO or MHO in the two cohorts, those in the MUNO or MUO groups were older, which indicates that older patients with digestive cancers may have poorer metabolic status. Medicare/Medicaid and private insurance were the main way of hospitalization payment for the majority of DSC patients. The distribution of median household income and residence of patients with different phenotypes showed no difference. As expected, participants with MUNO or MUO tended to have more common comorbidities, such as myocardial infarction, congestive heart failure, peripheral vascular disease, cerebrovascular disease, dementia, chronic pulmonary disease, connective tissue disease, ulcer disease, hemiplegia, moderate/severe renal disease, leukemia, lymphoma, and acquired immune deficiency syndrome (AIDS). Individuals with the MHNO phenotypes had significantly shorter LOS and fewer proportion of severe hospitalizations at index hospitalization compared with those with the MHO, MUNO, and MUO phenotypes (all *p* < 0.05). Compared with the MHO or MUO phenotypes, the MHNO and MUNO phenotypes accounted for a higher proportion of individuals with neoplasm of the upper digestive tract. Furthermore, the proportion of non-obese subjects in patients with neoplasm of the upper digestive tract was higher than that of obese subjects, regardless of metabolic status. Participants in the MUNO category had the highest proportion of patients with neoplasm of the liver and pancreas and the lowest proportion of patients with neoplasm of the lower digestive tract than those in the other three categories (**Table 1**).

**Table 1:** Baseline characteristics of the study population of digestive system cancer patients from the NRD, classified by different metabolic obesity phenotypes.

norm	30-day readmission cohort						180-day readmission cohort					
Variables	Total	MHNO	MHO	MUNO	MUO	*p*-value	Total	MHNO	MHO	MUNO	MUO	*p*-value
No. of cases	142,753	37,987	2,700	88,949	13,117		74,566	19,608	1,399	46,833	6,726	
Age (years), mean (SE)	67.16 ± 12.58	61.28 ± 13.27a	56.70 ± 12.30b	70.24 ± 11.34c	65.45 ± 10.99d	<0.001	67.38 ± 12.57	61.61 ± 13.29a	56.94 ± 12.47b	70.37 ± 11.39c	65.58 ± 10.84d	<0.001
Male, *n* (%)	82,298 (57.65)	21,476 (56.54)a	1,255 (46.48)b	52,619 (59.16)c	6,948 (52.97)d	<0.001	42,830 (57.44)	11,062 (56.42)a	658 (47.03)b	27,636 (59.01)c	3,474 (51.65)d	<0.001
Primary expected payer (%)						<0.001						<0.001
1. Medicare/Medicaid	97,547 (68.42)	21,034 (55.46)a	1,228 (45.52)b	66,584 (74.95)c	8,701 (66.39)d		51,643 (69.33)	11,073 (56.56)a	644 (46.10)b	35,429 (75.71)c	4,497 (66.91)d	
2. Private insurance	39,032 (27.38)	14,685 (38.72)a	1,318 (48.85)b	19,144 (21.51)c	3,915 (29.87)d		19,739 (26.50)	7,377 (37.68)a	686 (49.11)b	9,703 (20.73)c	1,973 (29.36)d	
3. Self-pay	2,424 (1.71)	1,042 (2.75)a	77 (2.85)a	1,123 (1.26)b	192 (1.46)b		1,232 (1.65)	522 (2.67)a	38 (2.72)a	586 (1.25)b	86 (1.28)b	
4. No charge/others	3,560 (2.50)	1,167 (3.08)a	75 (2.78)ab	2,020 (2.27)b	298 (2.27)b		1,879 (2.52)	606 (3.10)a	29 (2.08)ab	1,079 (2.31)b	165 (2.45)b	
Median household income						<0.001						<0.001
1. 0–25th percentile ($1–$37,999)	34,728 (24.68)	8,655 (23.14)a	657 (24.63)abc	22,020 (25.11)c	3,396 (26.23)b		18,273 (24.87)	4,433 (22.99)a	332 (23.99)ab	11,728 (25.40)b	1,780 (26.81)b	
2. 26th to 50th percentile ($38,000–$47,999)	37,759 (26.83)	9,866 (26.37)a	759 (28.46)ab	23,434 (26.73)a	3,700 (28.58)b		19,781 (26.92)	5,105 (26.47)a	395 (28.54)ab	12,352 (26.76)a	1,929 (29.06)b	
3. 51st to 75th percentile ($48,000– $63,999)	35,859 (25.49)	9,693 (25.91)a	704 (26.40)a	22,186 (25.30)a	3,276 (25.30)a		18,577 (25.28)	4,975 (25.80)a	352 (25.43)a	11,632 (25.20)a	1,618 (24.37)a	
4. 76th to 100th percentile ($64,000 or more)	32,359 (23.00)	9,193 (24.58)a	547 (20.51)b	20,043 (22.86)c	2,576 (19.89)b		16,842 (22.92)	4,771 (24.74)a	305 (22.04)abc	10,454 (22.64)c	1,312 (19.76)b	
Patient location (%)						<0.001						<0.001
1. “Central” counties of metro areas of ≥1 million population, *n* (%)	40,736 (28.61)	10,949 (28.93)a	668 (24.81)b	25,702 (28.96)a	3,417 (26.08)b		21,269 (28.60)	5,690 (29.13)a	333 (23.82)b	13,530 (28.96)a	1,716 (25.54)b	
2. “Fringe” counties of metro areas of ≥1 million population, *n* (%)	37,358 (26.24)	9,900 (26.15)a	678 (25.18)a	23,372 (26.34)a	3,408 (26.01)a		19,674 (26.45)	5,167 (26.45)a	357 (25.54)a	12,429 (26.60)a	1,721 (25.62)a	
3. Counties in metro areas of 250,000–999,999 population, *n* (%)	30,107 (21.14)	7,966 (21.04)a	621 (23.06)a	18,663 (21.03)a	2,857 (21.81)a		15,680 (21.08)	4,089 (20.93)ab	334 (23.89)b	9,774 (20.92)a	1,483 (22.08)ab	
4. Counties in metro areas of 50,000–249,999 population, *n* (%)	13,364 (9.39)	3,549 (9.38)a	273 (10.14)a	8,247 (9.29)a	1,295 (9.88)a		6,912 (9.29)	1,796 (9.20)a	137 (9.80)a	4,311 (9.23)a	668 (9.94)a	
5. Micropolitan counties, *n* (%)	11,601 (8.15)	3,091 (8.17)abc	256 (9.51)c	7,088 (7.99)b	1,166 (8.90)ac		6,019 (8.09)	1,600 (8.19)ab	135 (9.66)ab	3,675 (7.87)b	609 (9.07)a	
6. Not metropolitan or micropolitan counties, *n* (%)	9,221 (6.48)	2,398 (6.34)a	197 (7.32)ab	5,668 (6.39)a	958 (7.31)b		4,815 (6.47)	1,190 (6.09)a	102 (7.30)ab	3,002 (6.43)a	521 (7.76)b	
Deyo–Charlson comorbidity index (%) ≤2 >2 Length of stay (unadjusted by month of follow-up) Proportion with severe hospitalization (LOS >7 days) (%)	62,209 (43.58) 80,544 (56.42) 4 (3.8) 37,051 (25.95)	20,065 (52.82)a 17,922 (47.18)a 4 (3.7)a 8,908 (23.45)	1,386 (51.33)a 1,314 (48.67)a 5 (3.8)b 805 (29.81)	35,807 (40.26)b 53,142 (59.74)b 5 (3.8)c 23,374 (26.28)	4,951 (37.74)c 8,166 (62.26)c 5 (3.8)b 3,964 (30.22)	<0.001 <0.001 <0.001	31,359 (42.06) 43,207 (57.94) 5 (3.8) 19,603 (26.29)	10,002 (51.01)a 9,606 (48.99)a 4 (3.7)a 4,607 (23.50)a	726 (51.89)a 673 (48.10)a 5 (3.8)b 417 (29.81)bc	18,126 (38.70)b 28,707 (61.30)b 5 (3.8)b 12,545 (26.79)c	2,505 (37.24)b 4,221 (62.76)b 5 (3.8)b 2,034 (30.24)b	<0.001 <0.001 <0.001
Neoplasm of the upper digestive tract, *n* (%)	19,716 (13.81)	5,177 (13.63)a	267 (9.89)b	12,797 (14.39)c	1,475 (11.24)b	<0.001	10,876 (14.59)	2,823 (14.40)a	145 (10.36)b	7,057 (15.07)a	851 (12.65)b	<0.001
Neoplasm of the lower digestive tract, *n* (%)	76,300 (53.44)	22,259 (58.60)a	1,886 (69.85)b	44,210 (49.70)c	7,945 (60.57)d	<0.001	38,702 (51.90)	11,116 (56.69)a	958 (68.47)b	22,638 (48.34)c	3,990 (59.32)d	<0.001
Neoplasm of the liver, *n* (%)	21,355 (14.96)	5,232 (13.77)a	313 (11.59)b	13,991 (15.73)c	1,819 (13.87)a	<0.001	11,289 (15.14)	2,773 (14.14)a	164 (11.72)a	7,420 (15.84)b	932 (13.86)a	<0.001
Neoplasm of the pancreas, *n* (%)	26,425 (18.51)	5,566 (14.65)a	248 (9.19)b	18,674 (20.99)c	1,937 (14.77)a	<0.001	13,922 (18.67)	2,947 (15.03)a	135 (9.65)b	9,874 (21.08)c	966 (14.36)a	<0.001

The small letters (e.g. a, b, c, d, etc.) in this table refer to comparisons between groups. There is no statistical difference between groups with the same small letters.

MHNO, metabolically healthy non-obese; MHO, metabolically healthy obese; MUNO, metabolically unhealthy non-obese; MUO, metabolically unhealthy obese.

### 3.2 Short- and long-term readmission risk for different DSCs classified by obesity and metabolic health status

**Figure 2:**
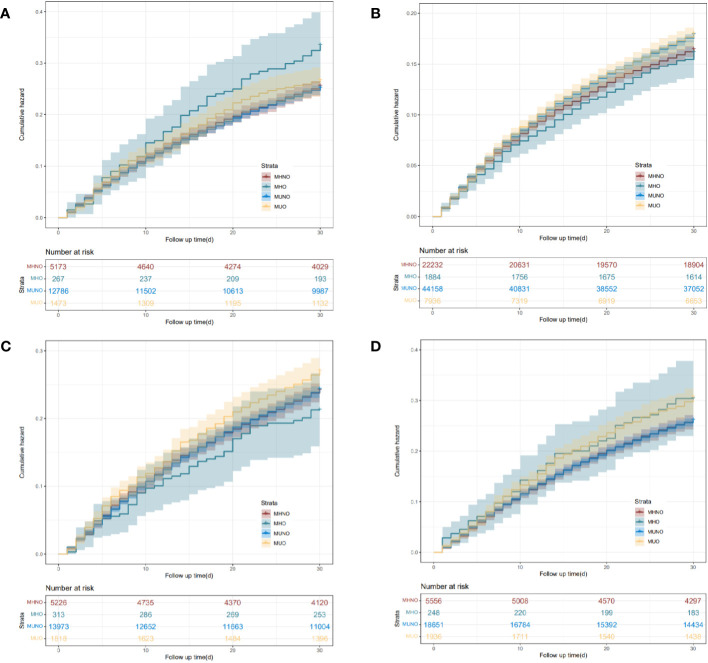
Kaplan–Meier curves of the 30-day readmission for the four phenotypes of different digestive system cancers. **(A)** Neoplasm of the upper digestive tract. **(B)** Neoplasm of the lower digestive tract. **(C)** Neoplasm of the liver. **(D)** Neoplasm of the pancreas. MHNO, metabolically healthy non-obese; MHO, metabolically healthy obese; MUNO, metabolically unhealthy non-obese; MUO, metabolically unhealthy obese.

Patients were followed over 26.63 ± 7.85 days after index hospitalization in the 30-day readmission cohort. For neoplasm of the upper digestive tract, the MHO phenotype had a significantly higher rate of readmission within 30 days (*p* < 0.01), as compared with the other three phenotypes (**Supplementary Table 2**, **Figure 2**). Compared with the MHNO phenotype, higher 30-day readmission rates were observed in the MUNO and MUO phenotypes in patients with neoplasm of the lower digestive tract. For neoplasm of the liver, there was no significant difference in readmission rates among the four phenotypes. The MUO phenotype in patients with neoplasm of the pancreas had a higher rate of 30-day readmission than the MHNO and MUNO phenotypes.

**Figure 3:**
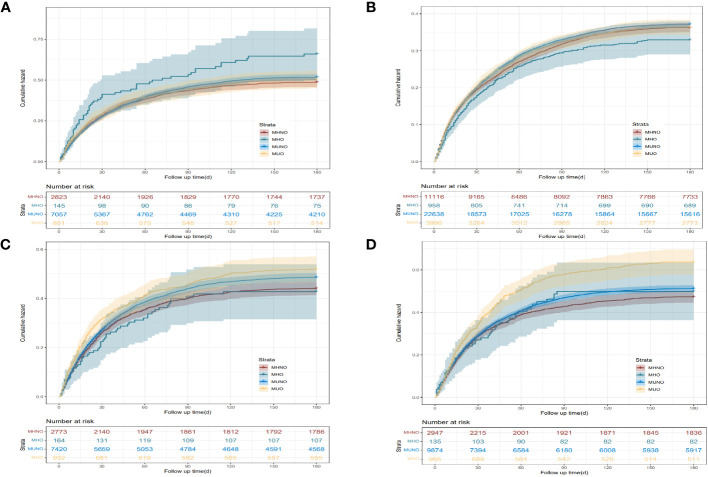
Kaplan–Meier curves of the 180-day readmission for the four phenotypes of different digestive system cancer. **(A)** Neoplasm of the upper digestive tract. **(B)** Neoplasm of the lower digestive tract. **(C)** Neoplasm of the liver. **(D)** Neoplasm of the pancreas. MHNO, metabolically healthy non-obese; MHO, metabolically healthy obese; MUNO, metabolically unhealthy non-obese; MUO, metabolically unhealthy obese.

Following up 129.18 ± 72.55 days, no difference was found in the long-term readmission rate among the four metabolic health statuses in patients with neoplasm of the upper digestive tract and lower digestive tract. However, unlike the short-term readmission rate, the long-term readmission rate of liver cancer patients with the MUO phenotype was higher than that of patients with the MHNO phenotype. For neoplasm of the pancreas, differences in long-term readmission rates among the four phenotypic patients were the same as the differences in short-term readmission rates (**Supplementary Table 2**, **Figure 3**).

On multivariable analysis, adjusting for age, sex, primary expected payer, patient location, Deyo–Charlson comorbidity index, LOS at index hospitalization, and severe hospitalization at index hospitalization, the MHO, MUNO, and MUO phenotypes were short- and long-term readmission risk factors of DSC compared with the MHNO phenotype (**Supplementary Table 3**).

Specifically, the MUNO phenotype had 1.147-fold (95% CI: 1.066, 1.235; *p* < 0.001) increased risks of the 180-day readmission in patients with neoplasm of the upper digestive tract (**Supplementary Table 3**). The MUNO phenotype had 1.073-fold (95% CI: 1.027, 1.121; *p* = 0.002) increased risks of the 30-day readmission (**Supplementary Table 3**) and 1.067-fold (*p* = 0.004) increased risks of the 180-day readmission (**Supplementary Table 3**) in patients with neoplasm of the lower digestive tract, whereas the MHO phenotype was a protective factor for the 180-day readmission in these patients with cancer [aHR, 0.873 (95% CI, 0.771, 0.989), *p* = 0.033]. The MUNO and MUO phenotypes were independent risk factors of short- and long-term readmission in patients with liver or pancreatic neoplasm (*p* < 0.05; **Supplementary Table 3**).

### 3.3 In-depth subgroup analysis of short- and long-term readmission risk

To explore the effect of obesity, hyperlipidemia, hyperglycemia, and hypertension on the short- or long-term readmission risk of DSC, we divided the study population into 10 groups for comparison. Consistently, no group was a risk factor for the 30-day readmission in patients with neoplasm of the upper digestive tract (**Supplementary Table 4**, **Supplementary Figure 1A**). Non-obese and simple hypertension abnormalities as well as non-obese and multiple metabolic abnormalities were risk factors for the 180-day readmission in these patients (**Supplementary Table 4**, **Supplementary Figure 2A**). For patients with neoplasm of the lower digestive tract, non-obese and simple hypertension abnormalities as well as non-obese and multiple metabolic abnormalities were risk factors for the 30-day readmission. Non-obese and simple hypertension abnormalities as well as non-obese and multiple metabolic abnormalities were risk factors for the 180-day readmission, while non-obese and simple hyperlipidemia abnormalities as well as obese and no metabolic abnormality were protective factors for the 180-day readmission (**Supplementary Table 4**, **Supplementary Figures 1B**, **2B**). Non-obese and simple hyperglycemia, non-obese and simple hyperlipidemia, non-obese and multiple metabolic abnormalities, and obese and multiple metabolic abnormalities were independent risk factors of short- and long-term readmission in patients with neoplasm of the liver (**Supplementary Table 4**, **Supplementary Figures 1C**, **2C**). Non-obese and multiple metabolic abnormalities as well as obese and multiple metabolic abnormalities were risk factors for the 30-day and 180-day readmission in patients with neoplasm of the pancreas (**Supplementary Table 4**, **Supplementary Figures 1D**, **2D**).

Furthermore, after grouping the patients according to the number of metabolic abnormalities, this independent impact of non-obese and two to three types of metabolic abnormality phenotypes on the risk of the 30-day and 180-day readmission was observed in patients with neoplasm of the lower digestive tract, liver, and pancreas. Also, being obese and the three types of metabolic abnormality phenotypes were risk factors for the 30-day and 180-day readmission in patients with neoplasm of the liver and pancreas (**Supplementary Table 5**, **Supplementary Figures 3**, **4**).

### 3.4 Secondary outcomes of patients with metabolic obesity phenotypes

For neoplasm of the upper digestive tract, the MUNO phenotype was not a risk factor for the 180-day severe hospitalization within 180 days but a risk factor for unplanned hospitalization within 180 days (**Supplementary Tables 6**, **7**).

Compared with the MHNO phenotype, higher 30-day readmission rates were shown in the MUNO and MUO phenotypes in patients with neoplasm of the lower digestive tract. Higher 30-day severe readmission rates were shown in the MUO phenotype in patients with neoplasm of the lower digestive tract compared with the MHO phenotype (**Supplementary Table 6**), while the 30-day unplanned hospitalization rate of the MUNO and MUO phenotypes was higher than that of the MHNO and MHO phenotypes in patients with neoplasm of the lower digestive tract (**Supplementary Table 2**).

For neoplasm of the liver, the MUNO and MUO phenotypes were risk factors for severe hospitalizations (**Supplementary Table 6**) and unplanned hospitalization within 30 days and 180 days compared with the MHNO phenotype (**Supplementary Table 7**).

For neoplasm of the pancreas, the MUNO and MUO phenotypes were risk factors for the 180-day severe hospitalizations (**Supplementary Table 6**) and the 30-day or 180-day unplanned hospitalization (**Supplementary Table 7**), compared with the MHNO phenotype.

### 3.5 Reasons for hospitalization by metabolic obesity status

**Figure 4:**
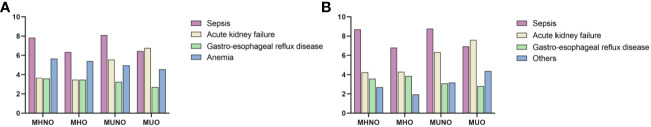
Reasons for hospitalization by metabolic obesity status. **(A)** The four most common diagnoses associated with the 30-day readmissions. **(B)** The four most common diagnoses associated with the 180-day readmissions. MHNO, metabolically healthy non-obese; MHO, metabolically healthy obese; MUNO, metabolically unhealthy non-obese; MUO, metabolically unhealthy obese.

In the top 4 diagnosis positions, the most frequent ICD-10 diagnoses for the 30-day readmissions are displayed in **Figure 4**. Concerning diagnoses not referencing DSC or metabolic obesity status, the top 4 most common diagnoses associated with the 30-day readmissions in the MHNO, MHO, and MUNO groups were sepsis, anemia, acute kidney failure, and gastroesophageal reflux disease. In contrast, in the MUO group, these diagnoses were acute kidney failure, sepsis, anemia, and gastroesophageal reflux disease (**Figure 4A**).

Notably, sepsis was the most common of the 180-day admission diagnoses not referencing DSC or metabolic obesity status in those with MHNO, MHO, and MUNO phenotypes. Acute kidney failure was the most common of these diagnoses in those with MUO. In addition, obstructive sleep apnea was one of the four main causes of the 180-day readmission in patients with MHO. Atherosclerotic heart disease was also the main cause of the 180-day readmissions for patients with the MUNO phenotype (**Figure 4B**).

## 4 Discussion

Based on this nationally representative longitudinal study using NRD, we made several crucial observations regarding the prognostic impact of metabolic obesity phenotypes in hospitalized individuals with four major categories of DSC. First, the MHO, MUNO, and MUO phenotypes were risk factors for the short- or long-term readmission of DSC compared with the MHNO phenotype, after adjustment for age, sex, and other confounding factors. More precisely, the effect of metabolism on the short- or long-term readmission of patients with liver or pancreas cancers may be stronger than that of obesity, highlighting the importance of abnormal metabolic status modification regardless of obesity status. Second, we observed that metabolic obesity status was independently associated with a significantly high risk of severe hospitalizations and unplanned hospitalization within 30 days or 180 days. Third, the most common non-tumor-related reason for readmission in DSC patients with the MHNO, MHO, and MUNO phenotypes was sepsis, whereas acute renal failure was the leading cause of the short- or long-term readmission for MUO phenotypes. Overall, this is the first large-scale study to systematically assess the impact of metabolic obesity status on short- and long-term clinical outcomes in patients with DSC, which might provide a guideline for the calibration of readmission risk stratification in these individuals.

Epidemiological data show the links between obesity and esophageal or gastric cancer (21). Results from the European Prospective Investigation into Cancer and Nutrition (EPIC) cohort with a mean follow-up of 14 years demonstrated that abdominal obesity was positively associated with upper gastrointestinal cancers (22). Although the high association between the two has been well studied, little is known regarding readmission rates in patients with metabolically defined obesity type. Our study fills the gap in knowledge of the effect of both obesity and metabolic abnormalities on readmission rates in patients with DSC. The 1.362-fold increased short-term readmission risk of the MHO phenotype among patients with neoplasm of the upper digestive tract in our multivariable models was both statistically and clinically significant, while other phenotypes had no significant effect on 30-day readmission in these patients. Moreover, the MUNO phenotype had 1.147-fold increased risks of long-term readmission in patients with neoplasm of the upper digestive tract, compared with the MHO, MHNO, and MUO phenotypes. These data suggest that obesity may have an important effect on short-term readmission in patients with neoplasm of the upper digestive tract, yet metabolic factors have a greater impact on long-term readmission in these patients. Our findings illustrate the need to improve the management and transitional care in patients with different metabolic obesity statuses.

Despite the association between obesity or BMI and patient outcomes after laparoscopic colorectal resection has been studied (23), whether metabolically defined obesity types affect the risk of rehospitalization of lower digestive tract tumors has not been systematically assessed. This undoubtedly brings excessive burden to clinical practice. Under this urgent situation, our retrospective cohort study revealed that metabolically unhealthy non-obese status was a significant risk factor for readmission in patients with lower digestive tract tumors, including the small intestine, large intestine, rectum, anal canal, and anus. One study previously demonstrated the detrimental effect of metabolic abnormalities on the prognosis of colorectal cancer, with significantly worse survival, recurrence, and liver metastases, suggesting that metabolic abnormalities are important prognostic factors for colorectal cancer (24). In addition, hypertension and high triglyceride levels were also identified as important risk factors for postoperative complications of colorectal cancer after surgery (25). These associations may be due to a multifactorial mechanism involving the action of inflammatory cytokines and adipokines, such as adiponectin, leptin, IGF-1, and others (26). Surprisingly, while obesity has been identified as a risk factor for multiple cancers (27), metabolically healthy obese status could reduce long-term readmission rates of patients with lower digestive tract tumors in our cohort. This phenomenon has been well described as the “obesity paradox” (28). We assume that progression of the cancer, such as cachexia and poor appetite, results in the inability to maintain body weight so that individuals with low BMI have higher rates of readmission, prolonged lengths of stay, and other worse prognosis.

We identified that liver or pancreas cancer patients with abnormal metabolic status were more likely to be readmitted to the hospital, independently of obesity status, highlighting that the effect of metabolic status on the development of liver or pancreas cancers may be stronger than that of obesity. Previous studies reported that all BMI categories in metabolically unhealthy individuals were associated with a high risk of liver cancer (17); the risk of liver cancer increased with the number of metabolic syndrome components in subjects not chronically infected with hepatitis viruses (29). Studies have found that lipids, which are metabolized primarily in the liver, play a crucial role in liver physiology and the pathological progression of diseases such as hepatocellular carcinoma. The progression of hepatocellular carcinoma is associated with inflammation and complex metabolic reprogramming. Drugs targeting lipid metabolism can interfere with lipid metabolism in the tumor and tumor microenvironment and become a new approach to the treatment of liver cancer. Liver cancer patients with hypertension had a high mortality (30). Structural activation of tyrosine kinases, cell overproliferation, and changes in growth factor receptors have been suggested as potential mechanisms by which hypertension may increase occurrence risk and poor prognosis of liver cancers (29, 31). Both prediabetes (32) and diabetes mellitus (33) are also found to be associated with high risks of liver cancers, which is consistent with our subgroup analysis of readmission risk. On the other hand, Chung HS et al. demonstrated that the metabolically unhealthy phenotypes (MUNO and MUO) significantly increased the risk of pancreatic cancer, whereas obese individuals with the metabolically healthy phenotypes did not (34). Chronic inflammation with changes in the concentration of inflammatory cytokines and the infiltration of pancreatic immunosuppressive cells can explain the association between metabolic abnormalities and the incidence and prognosis of pancreatic cancer (35), but more research is urgently needed to elucidate the underlying pathophysiological mechanisms. In combination with the metabolic obesity phenotype, clinical markers of liver or pancreatic cancer may effectively help screen patients at a high risk of readmission.

It has been estimated that patients with cancer have a 10-fold higher risk of sepsis than those without cancer (36), and cancer-related sepsis is associated with significantly increased mortality at all ages (37). We found that sepsis was the most common 180-day admission diagnosis that did not involve digestive cancer or metabolic obesity status among patients with MHNO, MHO, and MUNO phenotypes. Therefore, early recognition and improved management of sepsis may be beneficial to decrease the adverse outcomes, such as readmission and death in patients with digestive tract tumors. In addition, acute kidney failure was the most common of the readmission diagnoses in MUO individuals who participated in our studies. The interplay between cancer and acute kidney injury is intricate (38), and it should be remembered that acute kidney injury in a cancer setting is a serious complication of the disease course and clinicians should pay attention to the prevention and monitoring.

Several limitations should be noted in our study. First, the identification of digestive tract tumors and metabolic obesity status using ICD-10-CM diagnosis codes may be subject to potential misclassification. Second, this study is a retrospective cohort study design. Although we adjusted for potential confounders, the risk of unmeasured or unconsidered confounders that could affect the results cannot be excluded.

## 5 Conclusions

In summary, we observed that the MHO, MUNO, and MUO phenotypes were higher risk factors for poor prognosis, such as short- or long-term readmission, severe hospitalization, and unplanned hospitalization of patients with DSC, compared with those with the MHNO phenotype. The effect of metabolic status on the short- or long-term readmission of liver or pancreas cancer may be stronger than that of obesity, highlighting that clinical intervention should focus on not only obesity but also metabolic abnormalities. Sepsis and acute kidney injury were the leading causes of admission diagnosis in patients with DSC, independent of digestive cancer or metabolic obesity status. Future studies should focus on the identification of novel screening guidelines for the readmission risk of patients with DSC according to various metabolic obesity phenotypes.

## Data availability statement

The datasets presented in this study can be found in online repositories. The names of the repository/repositories and accession number(s) can be found in the article/**Supplementary Material**.

## Author contributions

JZ created the study protocol. XF contributed to the analysis plan. YC and YL wrote the first draft of the manuscript. XW, SS, YS, and HG revised the manuscript to create the final version. HG contributed to the design of the study protocol and reviewed the manuscript. XF contributed to the data analysis. All authors contributed to the article and approved the submitted version.

## Supplementary material

Supplementary Figure 1 Kaplan–Meier curves of the relationships between obesity, hyperglycemia, hyperlipidemia, and hypertension and the 30-day READMISSION of digestive system cancer in study population. **(A)** Neoplasm of upper digestive tract. **(B)** Neoplasm of lower digestive tract. **(C)** Neoplasm of liver. **(D)** Neoplasm of pancreas.

Supplementary Figure 2 Kaplan–Meier curves of the relationships between obesity, hyperglycemia, hyperlipidemia, and hypertension and the 180-day READMISSION of digestive system cancer in study population. **(A)** Neoplasm of upper digestive tract. **(B)** Neoplasm of lower digestive tract. **(C)** Neoplasm of liver. **(D)** Neoplasm of pancreas.

Supplementary Figure 3 Kaplan–Meier curves of the relationships between obesity with the number of metabolic abnormalities and the 30-day READMISSION of digestive system cancer in study population. **(A)** Neoplasm of upper digestive tract. **(B)** Neoplasm of lower digestive tract. **(C)** Neoplasm of liver. **(D)** Neoplasm of pancreas.

Supplementary Figure 4 Kaplan–Meier curves of the relationships between obesity with the number of metabolic abnormalities and the 180-day READMISSION of digestive system cancer in study population. **(A)** Neoplasm of upper digestive tract. **(B)** Neoplasm of lower digestive tract. **(C)** Neoplasm of liver. **(D)** Neoplasm of pancreas. MHNO, metabolically healthy nonobese; MHO, metabolically healthy obese; MUNO, metabolically unhealthy nonobese; MUO, metabolically unhealthy obese.
