# Supplementary figures and images for: The short- and long-term readmission of four major categories of digestive system cancers: does obesity or metabolic disorder matter?
Source: Front Endocrinol (Lausanne). 2023 Oct 30;14:1214651. doi: 10.3389/fendo.2023.1214651 (PMC10642772; doi:10.3389/fendo.2023.1214651)

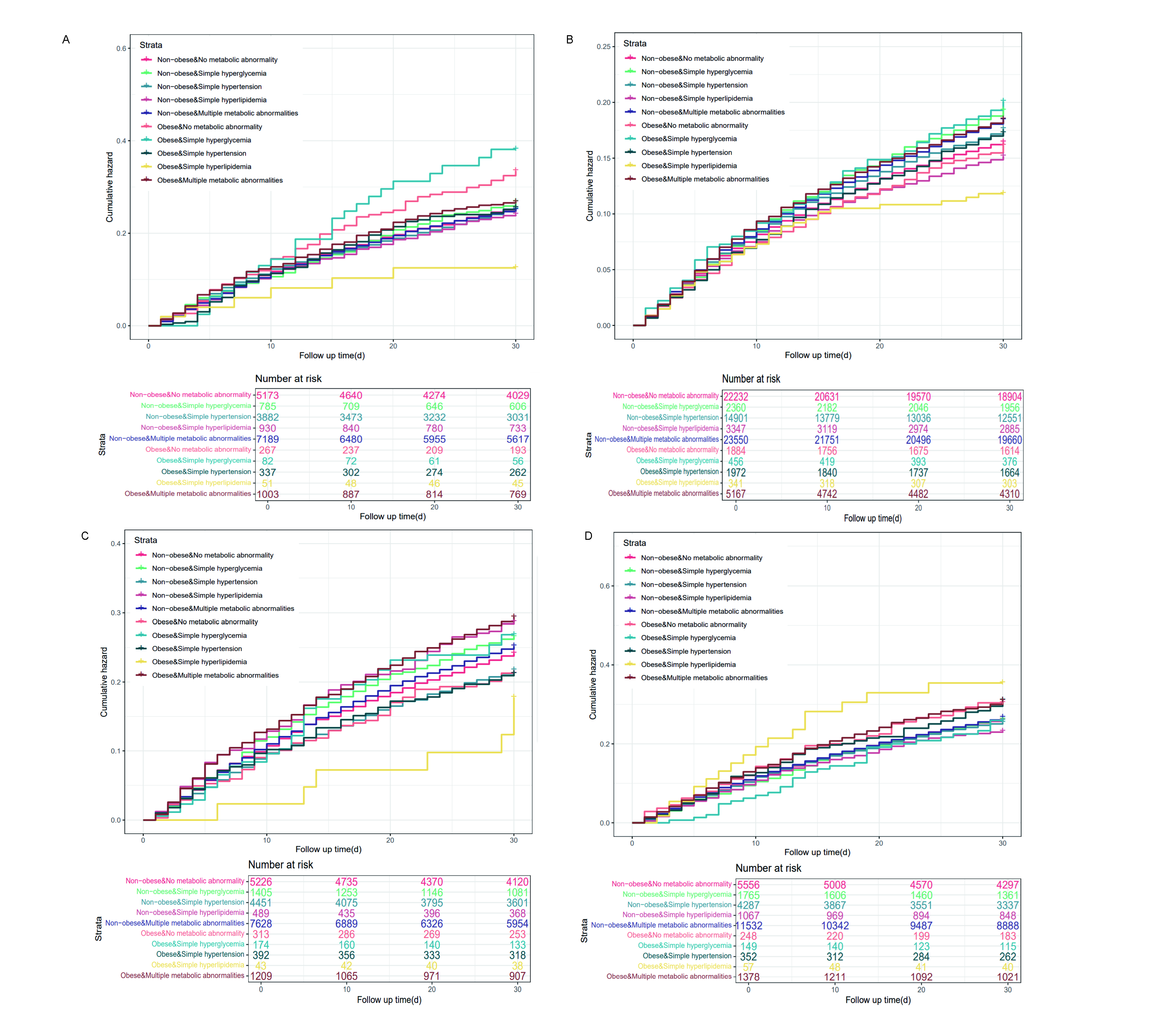

Supplement: Supplementary Figure 1 — Kaplan–Meier curves of the relationships between obesity, hyperglycemia, hyperlipidemia, and hypertension and the 30-day READMISSION of digestive system cancer in study population. (A) Neoplasm of upper digestive tract. (B) Neoplasm of lower digestive tract. (C) Neoplasm of liver. (D) Neoplasm of pancreas. [file Image_1.tiff]

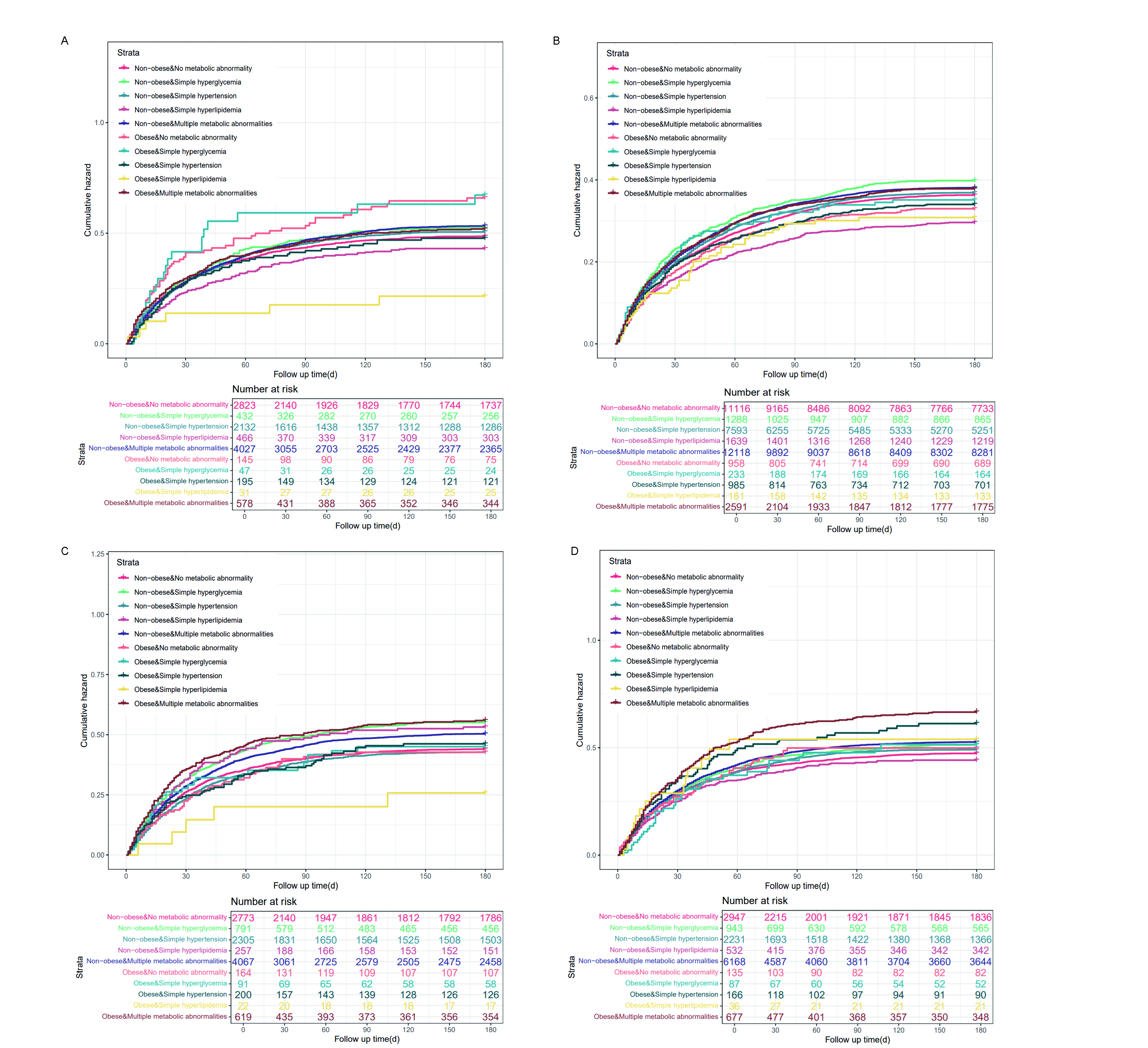

Supplement: Supplementary Figure 2 — Kaplan–Meier curves of the relationships between obesity, hyperglycemia, hyperlipidemia, and hypertension and the 180-day READMISSION of digestive system cancer in study population. (A) Neoplasm of upper digestive tract. (B) Neoplasm of lower digestive tract. (C) Neoplasm of liver. (D) Neoplasm of pancreas. [file Image_2.tiff]

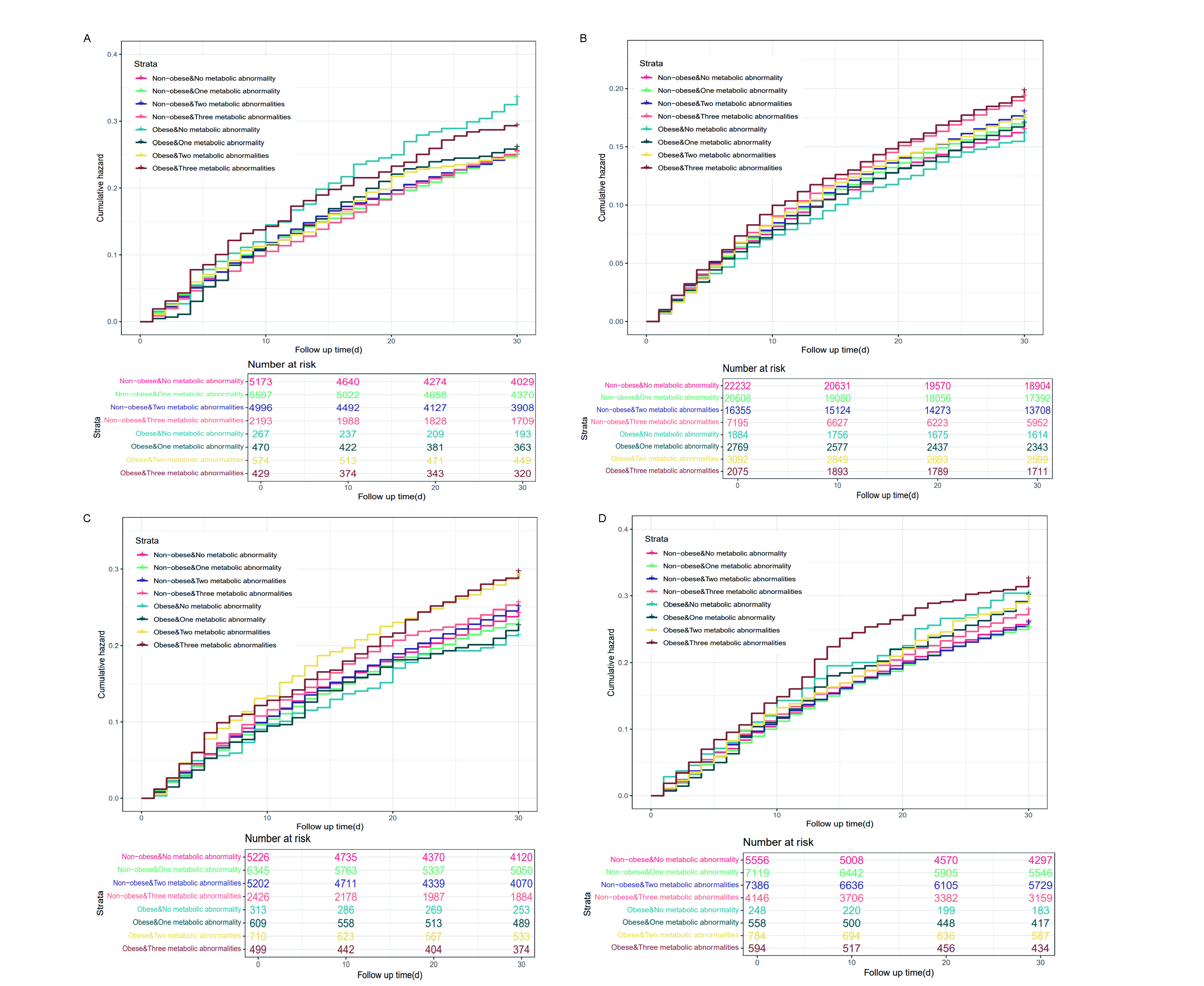

Supplement: Supplementary Figure 3 — Kaplan–Meier curves of the relationships between obesity with the number of metabolic abnormalities and the 30-day READMISSION of digestive system cancer in study population. (A) Neoplasm of upper digestive tract. (B) Neoplasm of lower digestive tract. (C) Neoplasm of liver. (D) Neoplasm of pancreas. [file Image_3.tif]

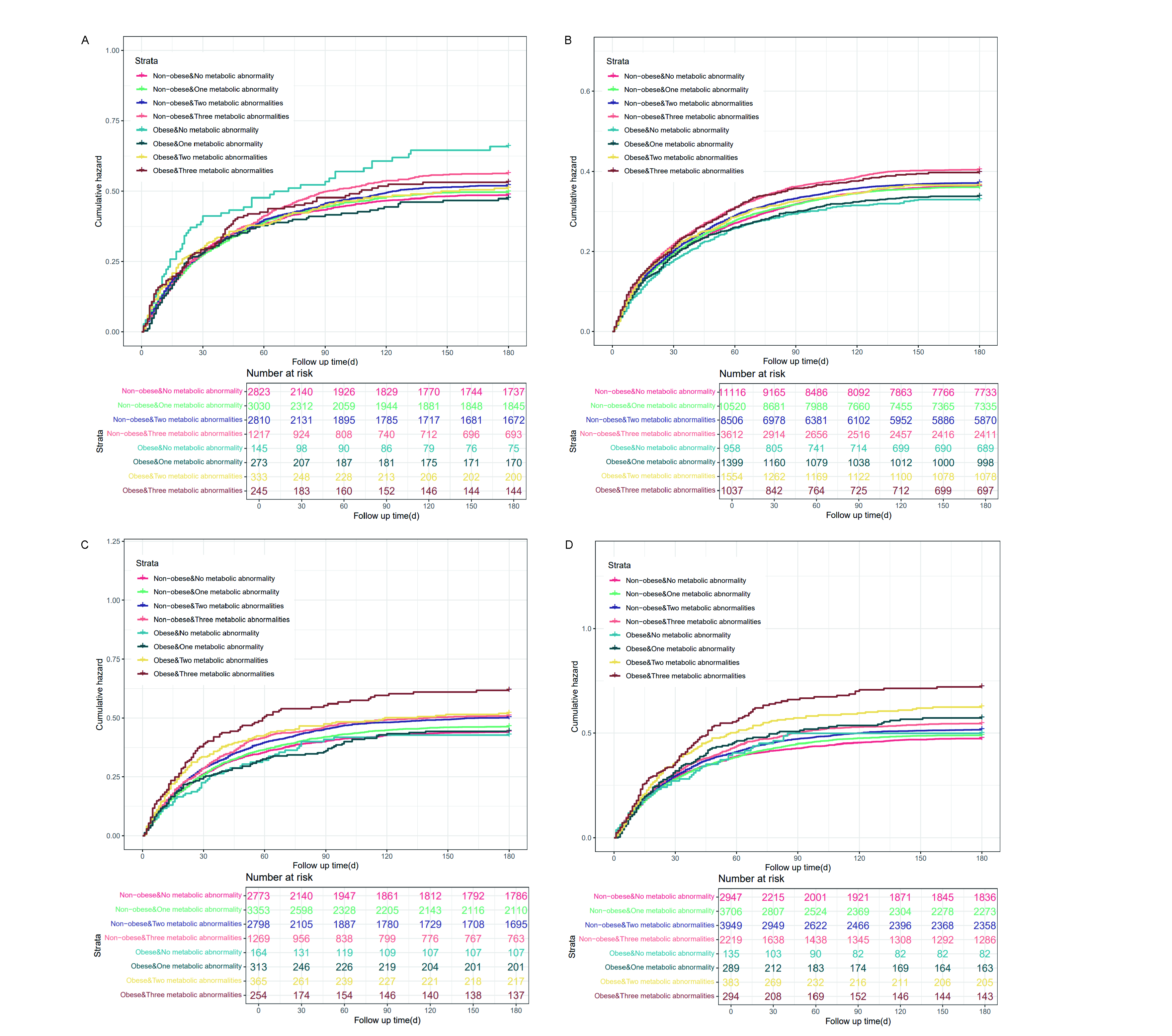

Supplement: Supplementary Figure 4 — Kaplan–Meier curves of the relationships between obesity with the number of metabolic abnormalities and the 180-day READMISSION of digestive system cancer in study population. (A) Neoplasm of upper digestive tract. (B) Neoplasm of lower digestive tract. (C) Neoplasm of liver. (D) Neoplasm of pancreas. MHNO, metabolically healthy nonobese; MHO, metabolically healthy obese; MUNO, metabolically unhealthy nonobese; MUO, metabolically unhealthy obese. [file Image_4.tif]
