# Supplementary material for: The short- and long-term readmission of four major categories of digestive system cancers: does obesity or metabolic disorder matter?
Source: Front Endocrinol (Lausanne). 2023 Oct 30;14:1214651. doi: 10.3389/fendo.2023.1214651 (PMC10642772; doi:10.3389/fendo.2023.1214651)
Supplement: Supplementary file 5 [file Table_1.docx]

**Supplementary Table 1**

| **Variables** | **ICD-10-CM diagnostic codes** |
| --- | --- |
| Neoplasm of upper digestive tract | C153-155,C158-C162,C164-C166,C168-C170 C7A092, D002, D131, D132,D371, D3A092 |
| Neoplasm of lower digestive tract | C171, C172, C178- C180, C182- C189, C19,C20, C210, C211 |
| Neoplasm of liver | C220- C224, C227- C229 |
| Neoplasm of pancreas | C250- C254, C257- C259 |
| Myocardial infarction | I2101, I2102, I2109, I2111, I2119, I2121, I2129, I213, I214, I219, I21A1, I21A9, I220, I221, I222, I228, I229 |
| Congestive heart failure | I5020- I5023, I5030- I5033, I5040- I5043 |
| Peripheral vascular disease | I70201- I70203, I70208- I70209, I70211- I70213, I70218, I70219, I70221- I70223, I70228- I70229, I70231- I70234 |
| Cerebrovascular disease | I60.x, I61.x, I62.x, I63.x, I65, I66.x, I67, I68, G46.x, |
| Dementia | F0150, F0151, F0280, F0281, F0390, F0391 |
| Chronic pulmonary disease | J44, J440, J441, J449 |
| Connective tissue disease | M30.x, M31.x, M32.x, M33.x, M34.x, M35.x, M36.x |
| Ulcer disease | K25.x, K26.x, K27.x, K28.x, K221.x, K626, K633 |
| Hemiplegia | G8100-G8104, G8110-G8114, G8190-G8194 |
| Moderate/Severe renal disease | N181,N182,N183,N1830,N1831,N1832,N184,N185,N186,N189 |
| Leukemia | C901.x, C91.x, C92.x, C93.x, C94.x, C95.x |
| Lymphoma | C81.x, C82.x, C83.x, C84.x, C85.x, C86.x, |
| Acquired immune deficiency syndrome | B20, B9735, Z21 |
| Prediabetes | R7303 |
| Diabetes | E0800-E089; E1010-E119 |
| Hyperlipidemia | E782; E784; E7849; E785 |
| Hypertension | I10.x; I110; I119; I120; I129; I130; I1310; I1311; I132; I150; I151; I152; I158; I159 |
| Obesity | Z6825- Z6829; E663; E669O; Z6830-Z6839; Z6841-Z6845; E6601; E662 |
| Sepsis | A400-A419 |
| Anemia | D500-D559 |
| Acute kidney failure | N17.x |
| Gastro-esophageal reflux disease | K210- K219 |

**Supplementary Table 2. Outcomes during follow–up in digestive system cancer patients with different metabolic obesity phenotypes.**

| **Variables** | **Total** | **MHNO** | **MHO** | **MUNO** | **MUO** | | **P-value** |
| --- | --- | --- | --- | --- | --- | --- | --- |
| **Days of follow-up in 30-day readmission cohort** | 26.63±7.85 | 26.76±7.73a | 26.89±7.57b | 26.58±7.88c | 26.50±7.97c | | <0.001 |
| **30-day Readmission (n, %)** |  |  |  |  |  | |  |
| **Total** | 27183(19.04) | 6883(18.12)a | 481(17.81)ab | 17247(19.39)b | 2572(19.61)b | | <0.001 |
| **Neoplasm of upper digestive tract** | 4435(22.49 | 1166(22.52)a | 76(28.46)a | 2849(22.26)a | 344(23.32)a | | 0.092 |
| **Neoplasm of lower digestive tract** | 12228(16.03) | 3374(15.16)a | 282(14.95)ab | 7264(16.43)b | 1308(16.46)b | | <0.001 |
| **Neoplasm of liver** | 4637(21.71) | 1124(21.48)a | 60(19.17)a | 3022(21.60)a | 431(23.69)a | | 0.129 |
| **Neoplasm of pancreas** | 6149(23.27) | 1277(22.94)a | 65(26.21)ab | 4295(23.0)a | 512(26.43)b | | 0.004 |
| **30-day Severe hospitalization (LOS >7 days)** |  |  |  |  |  | |  |
| **Total** | 7281(5.10) | 1833(4.83)a | 132(4.89)ab | 4585(5.15)ab | 731(5.57)b | | 0.005 |
| **Neoplasm of upper digestive tract** | 1387(7.04) | 363 (7.01)a | 29(10.86)a | 873(6.82)a | 122(8.27)a | | 0.016 |
| **Neoplasm of lower digestive tract** | 3264(4.28) | 915(4.11)a | 65(3.45)a | 1920(4.34)a | 364(4.58)a | | 0.074 |
| **Neoplasm of liver** | 1117(5.23) | 250(4.78)a | 18(5.75)ab | 731(5.23)ab | 118(6.49)b | | 0.044 |
| **Neoplasm of pancreas** | 1585(6.00) | 321(5.77)a | 21(8.47)a | 1111(5.95)a | 132(6.81)a | | 0.134 |
| **30-day Unplanned hospitalization (%)** |  |  |  |  |  | |  |
| **Total** | 23583(16.54) | 5913(15.58)a | 405(15.01)ab | 15029 (16.92)b | 2236(17.06)b | | <0.001 |
| **Neoplasm of upper digestive tract** | 3919(19.89) | 1026(19.83)a | 67(25.09)b | 2515(19.67)a | 311(21.11)ab | | 0.097 |
| **Neoplasm of lower digestive tract** | 10288(13.42) | 2811(12.64)a | 225(11.94)ab | 6099(13.81)b | 1093(13.77)ab | | <0.001 |
| **Neoplasm of liver** | 4246(19.90) | 1015(19.41)a | 54(17.25)a | 2784 (19.92)a | 393(21.62)a | | 0.138 |
| **Neoplasm of pancreas** | 5423(20.55) | 1111(20.00)a | 60(24.19)ab | 3794 (20.34)a | 458(23.66)b | | 0.002 |
| **Days of follow-up in 180-day readmission cohort** | 129.18±72.55 | 131.38±71.76a | 133.54±70.59a | 128.28±72.83b | 128.13±73.15b | | <0.001 |
| **180-day Readmission (n, %)** |  |  |  |  | |  |  |
| **Total** | 25858(34.68) | 6516(33.23)a | 446(31.88)ab | 16523(35.28)b | | 2373(35.28)b | <0.001 |
| **Neoplasm of upper digestive tract** | 4341(39.91) | 1086(38.47)a | 70(48.28)a | 2848(40.36)a | | 337(39.60)a | 0.063 |
| **Neoplasm of lower digestive tract** | 11891(30.72) | 3383(30.43)a | 269(28.08)a | 7022(31.02)a | | 1217(30.50)a | 0.203 |
| **Neoplasm of liver** | 4237(37.85) | 987(35.59)a | 57(34.76)ab | 2852(38.44)ab | | 377(40.45)b | 0.015 |
| **Neoplasm of pancreas** | 5576(40.05) | 1111(37.70)a | 53(39.26)ab | 3957(40.07)a | | 455(47.10)b | <0.001 |
| **180-day** **Severe hospitalization (LOS >7 days)** |  |  |  |  | |  |  |
| **Total** | 6186(8.30) | 1554(7.93)a | 111(7.93)a | 3942(8.42)a | | 579(8.61)a | 0.136 |
| **Neoplasm of upper digestive tract** | 1261(11.59) | 321(11.37)a | 23(15.86)a | 807(11.44)a | | 110(12.93)a | 0.225 |
| **Neoplasm of lower digestive tract** | 2731(7.06) | 787(7.08)a | 56(5.85)a | 1617(7.14)a | | 271(6.79)a | 0.418 |
| **Neoplasm of liver** | 927(8.21) | 204(7.36)a | 17(10.37)a | 614(8.27)a | | 92(9.87)a | 0.067 |
| **Neoplasm of pancreas** | 1328(9.54) | 257(8.72)a | 16(11.85)a | 945(9.57)a | | 110(11.39)a | 0.073 |
| **180-day** **Unplanned hospitalization (%)** |  |  |  |  | |  |  |
| **Total** | 21693(29.09) | 5351(27.29)a | 352(25.16)a | 14031(29.96)b | | 1959(29.13)b | <0.001 |
| **Neoplasm of upper digestive tract** | 3723(34.23) | 927(32.84)a | 56(38.62)a | 2458(34.83)a | | 282(33.14)a | 0.154 |
| **Neoplasm of lower digestive tract** | 9387(24.25) | 2616(23.53)ab | 196(20.46)b | 5624(24.84)a | | 951(23.83)ab | 0.002 |
| **Neoplasm of liver** | 3892(34.48) | 895(32.28)a | 54(32.93)ab | 2597(35.00)ab | | 346(37.12)b | 0.019 |
| **Neoplasm of pancreas** | 4884(35.08) | 958(32.51)a | 48(35.56)abc | 3488(35.33)c | | 390(40.37)b | <0.001 |

The small letters (e.g. a, b, c, d, etc) in this table refer to comparisons between groups. There is no statistical difference between groups with the same small letters. Abbreviations: MHNO, metabolically healthy nonobese; MHO, metabolically healthy obese; MUNO, metabolically unhealthy nonobese; MUO, metabolically unhealthy obese.

**Supplementary Table 3 Cox proportional hazard analysis evaluating risk of READMISSION in digestive system cancer patients.**

|  | | **30-day Readmission** | | | | | | |  | **180-day Readmission** | | | | | | | | | | | |
| --- | --- | --- | --- | --- | --- | --- | --- | --- | --- | --- | --- | --- | --- | --- | --- | --- | --- | --- | --- | --- | --- |
| **Variables** | **HR (95% CI)-neoplasm of upper digestive tract** | **P-value**  **1** | **HR (95% CI)-neoplasm of lower digestive tract** | **P-value**  **2** | **HR (95% CI)-neoplasm of liver** | **P-value 3** | **HR (95% CI)-neoplasm of pancreas** | **P-value**  **4** |  | **HR (95% CI)-neoplasm of upper digestive tract** | **P-value**  **1** | **HR (95% CI)-neoplasm of lower digestive tract** | **P-value**  **2** | | **HR (95% CI)-neoplasm of liver** | **P-value 3** | **HR (95% CI)-neoplasm of pancreas** | | **P-value**  **4** | |  |
| **Metabolic obesity phenotypes** |  |  |  |  |  |  |  |  |  |  |  |  |  | |  |  |  |  | |  | |
| **MHNO** | 1(ref) |  | 1(ref) |  | 1(ref) |  | 1(ref) |  |  | 1(ref) |  | 1(ref) |  | | 1(ref) |  |  | 1(ref) | |  | |
| **MHO** | 1.244 (0.986,1.570) | 0.066 | 0.951(0.841,1.074) | 0.415 | 0.846(0.651,1.099) | 0.210 | 1.101(0.858,1.413) | 0.450 |  | 1.273(0.999,1.623) | 0.051 | 0.873(0.771,0.989) | 0.033 | | 0.968(0.739,1.267) | 0.811 |  | 0.991(0.752,1.306) | | 0.946 | |
| **MUNO** | 1.055(0.982,1.134) | 0.142 | 1.073(1.027,1.121) | 0.002 | 1.073(0.999,1.153) | 0.054 | 1.104(1.035,1.179) | 0.003 |  | 1.147(1.066,1.235) | <0.001 | 1.067(1.021,1.115) | 0.004 | | 1.190(1.103,1.283) | <0.001 |  | 1.206(1.125,1.292) | | <0.001 | |
| **MUO** | 1.071(0.949,1.209) | 0.269 | 1.034(0.969,1.103) | 0.311 | 1.152(1.029,1.289) | 0.014 | 1.209(1.090,1.340) | <0.001 |  | 1.085(0.959,1.227) | 0.195 | 0.988(0.925,1.056) | 0.724 | | 1.274(1.129,1.437) | <0.001 |  | 1.395(1.250,1.556) | | <0.001 | |
| **Age (per 1y increase)** | 0.987(0.984,0.990) | <0.001 | 0.992(0.990,0.993) | <0.001 | 0.989(0.986,0.991) | <0.001 | 0.983(0.980,0.985) | <0.001 |  | 0.985(0.982,0.988) | <0.001 | 0.986(0.985,0.988) | <0.001 | | 0.985(0.982,0.988) | <0.001 |  | 0.980(0.978,0.983） | | <0.001 | |
| **Sex (women vs men)** | 0.972(0.910,1.039) | 0.405 | 0.954(0.921,0.989) | 0.011 | 1.007(0.946,1.072) | 0.825 | 0.974(0.926,1.025) | 0.308 |  | 0.967(0.905,1.033) | 0.320 | 0.965(0.931,1.001) | 0.057 | | 1.012(0.948,1.081) | 0.721 |  | 0.975(0.925,1.028) | | 0.354 | |
| **Primary expected payer** |  |  |  |  |  |  |  |  |  |  |  |  |  | |  |  |  |  | |  | |
| **4. No charge/others** | 1(ref) |  | 1(ref) |  | 1(ref) |  | 1(ref) |  |  | 1(ref) |  | 1(ref) |  | | 1(ref) |  |  | 1(ref) | |  | |
| **1. Medicare/Medicaid** | 0.995(0.834,1.186) | 0.952 | 1.210(1.067,1.371) | 0.003 | 1.281(1.070,1.535) | 0.007 | 1.090(0.917,1.295) | 0.328 |  | 1.242(1.028,1.499) | 0.024 | 1.159(1.028,1.306) | 0.016 | | 1.417(1.155,1.738) | 0.001 |  | 1.058(0.884,1.265) | | 0.539 | |
| **2. Private insurance** | 0.829(0.692,0.994) | 0.042 | 0.993(0.874,1.128) | 0.914 | 1.200(0.996,1.447) | 0.056 | 1.012(0.849,1.206) | 0.895 |  | 1.030(0.849,1.250) | 0.767 | 0.943(0.834,1.065) | 0.341 | 1.233(0.998,1.523) | | 0.052 |  | 0.929(0.774,1.115) | | 0.430 | |
| **3. Self-pay** | 0.781(0.582,1.047) | 0.098 | 1.029(0.860,1.232) | 0.755 | 0.990(0.736,1.322) | 0.948 | 1.044(0.801,1.361) | 0.750 |  | 0.895(0.658,1.219) | 0.483 | 0.971(0.813,1.158) | 0.740 | 0.914(0.653,1.279) | | 0.600 |  | 1.093(0.829,1.442) | | 0.527 | |
| **Patient Location** | 0.960(0.941,0.980) | <0.001 | 0.984(0.972,0.996) | 0.007 | 0.960(0.940,0.980) | <0.001 | 0.956(0.939,0.973) | <0.001 |  | 0.954(0.935,0.973) | <0.001 | 0.975(0.963,0.987) | <0.001 | 0.936(0.916,0.956) | | <0.001 |  | 0.939(0.922,0.957) | | <0.001 | |
| **Deyo-Charlson Comorbidity Index** | 1.180 (1.102,1.262) | <0.001 | 1.501(1.444,1.561) | <0.001 | 1.144(1.068,1.226) | <0.001 | 1.096(1.036,1.159) | 0.001 |  | 1.095(1.024,1.171) | 0.008 | 1.494(1.436,1.554) | <0.001 | 1.046(0.974,1.122) | | 0.215 |  | 0.953(0.899,1.010) | | 0.105 | |
| **Length of stay at index hospitalization (per 1-day increase)** | 0.999(0.994,1.003) | 0.559 | 1.000(0.997,1.003) | 0.862 | 1.001(0.995,1.006) | 0.789 | 0.994(0.988,1.000) | 0.042 |  | 0.998(0.993,1.003) | 0.398 | 0.998(0.995,1.001) | 0.126 | 1.001(0.995,1.007) | | 0.699 |  | 0.994(0.988,1.000) | | 0.065 | |
| **Severe hospitalization at index hospitalization** | 0.975(0.897,1.059) | 0.542 | 1.130(1.074,1.188) | <0.001 | 1.045(0.956,1.143) | 0.332 | 1.039(0.956,1.130) | 0.364 |  | 0.859(0.789,0.936) | 0.001 | 1.128(1.071,1.188) | <0.001 | 0.851(0.773,0.936) | | 0.001 |  | 0.966(0.884,1.055) | | 0.440 | |

Abbreviations: MHNO, metabolically healthy nonobese; MHO, metabolically healthy obese; MUNO, metabolically unhealthy nonobese; MUO, metabolically unhealthy obese. HR: hazard ratios.

**Supplementary Table 4 Cox proportional hazard Analysis of the relationships between obesity, hyperglycemia, hyperlipidemia, and hypertension and the READMISSION of digestive system cancer in study population.**

| **Variables** | **Non-obese** | | | | |  | **Obese** | | | | |
| --- | --- | --- | --- | --- | --- | --- | --- | --- | --- | --- | --- |
|  | **No metabolic abnormality** | **Simple hyperglycemia** | **Simple hypertension** | **Simple hyperlipidemia** | **Multiple metabolic abnormalities** |  | **No metabolic abnormality** | **Simple hyperglycemia** | **Simple hypertension** | **Simple hyperlipidemia** | **Multiple metabolic abnormalities** |
| **30-day Readmission** |  |  |  |  |  |  |  |  |  |  |  |
| **HR (95% CI)-****neoplasm of upper digestive tract** | 1(ref) | 1.052 (0.900,1.231) | 1.058(0.967,1.158) | 0.998(0.857,1.162) | 1.064(0.982,1.153) |  | 1.244(0.985,1569) | 1.423(0.964,2.099) | 1.005(0.797,1.268) | 0.498(0.223,1.112) | 1.097(0.953,1.263) |
| **P-value 1** |  | 0.523 | 0.222 | 0.979 | 0.132 |  | 0.066 | 0.076 | 0.967 | 0.089 | 0.199 |
| **HR (95% CI)-neoplasm of lower digestive tract** | 1(ref) | 1.099(0.992,1.217) | 1.061(1.006,1.120) | 0.974(0.883,1.073) | 1.096(1.043,1.152) |  | 0.950(0.841,1.073) | 1.089(0.876,1.355) | 0.991(0.882,1.113) | 0.766(0.556,1.054) | 1.064(0.987,1.147) |
| **P-value 2** |  | 0.071 | 0.031 | 0.590 | <0.001 |  | 0.407 | 0.442 | 0.877 | 0.102 | 0.107 |
| **HR (95% CI)-neoplasm of liver** | 1(ref) | 1.124(0.993,1.272) | 0.956(0.873,1.046) | 1.283(1.063,1.548) | 1.130(1.043,1.224) |  | 0.846(0.651,1.099) | 1.085(0.794,1.483) | 0.905(0.716,1.143) | 0.619(0.277,1.380) | 1.275(1.122,1.449) |
| **P-value 3** |  | 0.065 | 0.324 | 0.009 | 0.003 |  | 0.210 | 0.607 | 0.402 | 0.241 | <0.001 |
| **HR (95% CI)-neoplasm of pancreas** | 1(ref) | 1.022(0.917,1.143) | 1.077(0.989,1.173) | 0.984(0.853,1.136) | 1.148(1.070,1.230) |  | 1.098(0.856,1.410) | 0.938(0.667,1.320) | 1.186(0.961,1.462) | 1.353(0.839,2.185) | 1.245(1.108,1.400) |
| **P-value 4** |  | 0.707 | 0.087 | 0.827 | <0.001 |  | 0.461 | 0.715 | 0.111 | 0.215 | <0.001 |
| **180-day Readmission** |  |  |  |  |  |  |  |  |  |  |  |
| **HR (95% CI)-neoplasm of upper digestive tract** | 1(ref) | 1.108(0.943,1.301) | 1.131(1.032,1.240) | 0.939(0.795,1.109) | 1.194(1.101.1.294) |  | 1.269(0.996,1.617) | 1.476(0.976,2.232) | 0.972(0.767,1.232) | 0.463(0.207,1.032) | 1.139(0.988,1.313) |
| **P-value 1** |  | 0.213 | 0.009 | 0.456 | <0.001 |  | 0.054 | 0.065 | 0.816 | 0.060 | 0.074 |
| **HR (95% CI)-neoplasm of lower digestive tract** | 1(ref) | 1.068(0.965,1.183) | 1.058(1.002,1.117) | 0.902(0.814,0.999) | 1.103(1.049,1.159) |  | 0.871(0.769,0.987) | 0.893(0.703,1.133) | 0.916(0.811,1.034) | 0.912(0.686,1.212) | 1.034(0.957,1.118) |
| **P-value 2** |  | 0.202 | 0.041 | 0.047 | <0.001 |  | 0.030 | 0.352 | 0.155 | 0.525 | 0.392 |
| **HR (95% CI)-neoplasm of liver** | 1(ref) | 1.281(1.130,1.453) | 1.039(0.944,1.142) | 1.293(1.056,1.583) | 1.266(1.164,1.377) |  | 0.969(0.740,1.269) | 1.083(0.765,1.533) | 1.120(0.883,1.419) | 0.493(0.185,1.316) | 1.400(1.220,1.607) |
| **P-value 3** |  | <0.001 | 0.435 | 0.013 | <0.001 |  | 0.816 | 0.654 | 0.349 | 0.158 | <0.001 |
| **HR (95% CI)-neoplasm of pancreas** | 1(ref) | 1.112(0.990,1.251) | 1.158(1.058,1.268) | 1.011(0.866,1.181) | 1.269(1.179,1.366) |  | 0.986(0.748,1.300) | 1.045(0.746,1.463) | 1.288(1.021,1.626) | 1.169(0.702,1.946) | 1.494(1.320,1.691) |
| **P-value 4** |  | 0.074 | 0.001 | 0.888 | <0.001 |  | 0.923 | 0.799 | 0.033 | 0.549 | <0.001 |

Abbreviations: HR: hazard ratios.

**Supplementary Table 5 Cox proportional hazard Analysis of the relationships between different combinations of nonobesity or obesity combined with metabolic abnormalities and the READMISSION of digestive system cancer in the study population.**

| **Variables** | **Non-obese** | | | | |  | **Obese** | | | | |
| --- | --- | --- | --- | --- | --- | --- | --- | --- | --- | --- | --- |
|  | **No metabolic abnormality** | **One metabolic abnormality** | **Two metabolic abnormalities** |  | **Three metabolic abnormalities** |  | **No metabolic abnormality** | **One metabolic abnormality** | **Two metabolic abnormalities** |  | **Three metabolic abnormalities** |
| **30-day Readmission** |  |  |  |  |  |  |  |  |  |  |  |
| **HR (95% CI)-neoplasm of upper digestive tract** | 1(ref) | 1.047(0.965,1.137) | 1.066(0.977,1.162) |  | 1.060(0.951,1.183) |  | 1.243(0.985,1.569) | 1.019(0.837,1.242) | 1.024(0.852,1.231) |  | 1.196(0.982,1.457) |
| **P-value 1** |  | 0.268 | 0.149 |  | 0.292 |  | 0.067 | 0.849 | 0.799 |  | 0.075 |
| **HR (95% CI)-neoplasm of lower digestive tract** | 1(ref) | 1.053(1.002,1.106) | 1.086(1.029,1.146) |  | 1.120(1.047,1.198) |  | 0.949(0.840,1.072) | 0.982(0.888,1.086) | 1.027(0.934,1.129) |  | 1.118(1.004,1.246) |
| **P-value 2** |  | 0.043 | 0.003 |  | 0.001 |  | 0.402 | 0.725 | 0.581 |  | 0.042 |
| **HR (95% CI)-neoplasm of liver** | 1(ref) | 1.019(0.939,1.105) | 1.117(1.025,1.217) |  | 1.165(1.048,1.296) |  | 0.845(0.650,1.098) | 0.936(0.776,1.129) | 1.261(1.076,1.478) |  | 1.298(1.080,1.561) |
| **P-value 3** |  | 0.655 | 0.012 |  | 0.005 |  | 0.208 | 0.487 | 0.004 |  | 0.006 |
| **HR (95% CI)-neoplasm of pancreas** | 1(ref) | 1.049(0.973,1.130) | 1.118(1.037,1.206) |  | 1.200(1.102,1.307) |  | 1.098(0.856,1.410) | 1.132(0.953,1.345) | 1.189(1.024,1.379) |  | 1.323(1.124,1.558) |
| **P-value 4** |  | 0.213 | 0.004 |  | <0.001 |  | 0.462 | 0.157 | 0.023 |  | 0.001 |
| **180-day Readmission** |  |  |  |  |  |  |  |  |  |  |  |
| **HR (95% CI)-neoplasm of upper digestive tract** | 1(ref) | 1.097(1.008,1.194) | 1.170(1.073,1.277) |  | 1.250(1.121,1.393) |  | 1.269(0.995,1.617) | 0.984(0.803,1.206) | 1.125(0.939,1.348) |  | 1.158(0.943,1.421) |
| **P-value 1** |  | 0.032 | <0.001 |  | <0.001 |  | 0.055 | 0.877 | 0.202 |  | 0.162 |
| **HR (95% CI)-neoplasm of lower digestive tract** | 1(ref) | 1.036(0.985,1.089) | 1.087(1.029,1.147) |  | 1.140(1.064,1.221) |  | 0.871(0.769,0.987) | 0.911(0.821,1.011) | 1.006(0.913,1.108) |  | 1.077(0.963,1.206) |
| **P-value 2** |  | 0.166 | 0.003 |  | <0.001 |  | 0.030 | 0.078 | 0.909 |  | 0.195 |
| **HR (95% CI)-neoplasm of liver** | 1(ref) | 1.116(1.024,1.215) | 1.260(1.152,1.378) |  | 1.291(1.155,1.443) |  | 0.968(0.739,1.268) | 1.061(0.871,1.292) | 1.293(1.086,1.539) |  | 1.572(1.296,1.908) |
| **P-value 3** |  | 0.012 | <0.001 |  | <0.001 |  | 0.812 | 0.556 | 0.004 |  | <0.001 |
| **HR (95% CI)-neoplasm of pancreas** | 1(ref) | 1.125(1.038,1.218) | 1.238(1.143,1.340) |  | 1.326(1.212,1.452) |  | 0.986(0.748,1.300) | 1.196(0.994,1.439) | 1.404(1.198,1.645) |  | 1.617(1.363,1.918) |
| **P-value 4** |  | 0.004 | <0.001 |  | <0.001 |  | 0.922 | 0.058 | <0.001 |  | <0.001 |

Abbreviations: HR: hazard ratios.

**Supplementary Table 6 Cox proportional hazard analysis evaluating risk of Severe Hospitalization in digestive system cancer patients.**

|  | | **30-day Severe Hospitalization** | | | | | | |  | **180-day Severe Hospitalization** | | | | | | | | | | | |
| --- | --- | --- | --- | --- | --- | --- | --- | --- | --- | --- | --- | --- | --- | --- | --- | --- | --- | --- | --- | --- | --- |
| **Variables** | **HR (95% CI)-neoplasm of upper digestive tract** | **P-value**  **1** | **HR (95% CI)-neoplasm of lower digestive tract** | **P-value**  **2** | **HR (95% CI)-neoplasm of liver** | **P-value 3** | **HR (95% CI)-neoplasm of pancreas** | **P-value**  **4** |  | **HR (95% CI)-neoplasm of upper digestive tract** | **P-value**  **1** | **HR (95% CI)-neoplasm of lower digestive tract** | **P-value**  **2** | | **HR (95% CI)-neoplasm of liver** | **P-value 3** | **HR (95% CI)-neoplasm of pancreas** | | **P-value**  **4** | |  |
| **Metabolic obesity phenotypes** |  |  |  |  |  |  |  |  |  |  |  |  |  | |  |  |  |  | |  | |
| **MHNO** | 1(ref) |  | 1(ref) |  | 1(ref) |  | 1(ref) |  |  | 1(ref) |  | 1(ref) |  | | 1(ref) |  |  | 1(ref) | |  | |
| **MHO** | 1.468(1.005,2.145) | 0.047 | 0.795(0.618,1.023) | 0.074 | 1.116(0.691,1.803) | 0.653 | 1.335(0.857,2.078) | 0.201 |  | 1.369(0.895,2.094) | 0.147 | 0.770(0.586,1.010) | 0.059 | | 1.378(0.839,2.262) | 0.206 |  | 1.218(0.734,2.021) | | 0.445 | |
| **MUNO** | 1.077(0.946,1.226) | 0.263 | 1.005(0.924,1.093) | 0.091 | 1.173(1.009,1.363) | 0.038 | 1.136(0.999,1.293) | 0.052 |  | 1.143(0.997,1.310) | 0.056 | 1.019(0.930,1.116) | 0.684 | | 1.219(1.034,1.438) | 0.018 |  | 1.244(1.079,1.435) | | 0.003 | |
| **MUO** | 1.229(0.999,1.512) | 0.051 | 1.022(0.904,1.155) | 0.732 | 1.387(1.110,1.733) | 0.004 | 1.211(0.987,1.485) | 0.066 |  | 1.215(0.977,1.511) | 0.080 | 0.913(0.795,1.050) | 0.203 | | 1.481(1.152,1.903) | 0.002 |  | 1.407(1.124,1.761) | | 0.003 | |
| **Age (per 1y increase)** | 0.985(0.980,0.990) | <0.001 | 0.993(0.990,0.997) | <0.001 | 0.987(0.982,0.993) | <0.001 | 0.983(0.978,0.988) | <0.001 |  | 0.983(0.978,0.988) | <0.001 | 0.985(0.982,0.988) | <0.001 | | 0.984(0.978,0.991) | <0.001 |  | 0.981(0.976,0.987) | | <0.001 | |
| **Sex (women vs men)** | 1.067(0.950,1.198) | 0.273 | 0.983(0.917,1.054) | 0.628 | 1.100(0.970,1.247) | 0.137 | 1.020(0.924,1.127) | 0.690 |  | 1.014(0.898,1.144) | 0.826 | 0.974(0.903,1.051) | 0.493 | | 1.018(0.884,1.172) | 0.804 |  | 1.000(0.897,1.115) | | 0.995 | |
| **Primary expected payer** |  |  |  |  |  |  |  |  |  |  |  |  |  | |  |  |  |  | |  | |
| **4. No charge/others** | 1(ref) |  | 1(ref) |  | 1(ref) |  | 1(ref) |  |  | 1(ref) |  | 1(ref) |  | | 1(ref) |  |  | 1(ref) | |  | |
| **1. Medicare/Medicaid** | 0.975(0.714,1.332) | 0.872 | 1.362(1.050,1.767) | 0.020 | 2.107(1.318,3.367) | 0.002 | 0.989(0.713,1.370) | 0.945 |  | 1.220(0.860,1.732) | 0.265 | 1.432(1.090,1.881) | 0.010 | | 1.956(1.171,3.269) | 0.010 |  | 0.846(0.604,1.186) | | 0.332 | |
| **2. Private insurance** | 0.799(0.580,1.101) | 0.170 | 1.163(0.893,1.516) | 0.263 | 1.921(1.189,3.105) | 0.008 | 0.927(0.665,1.294) | 0.656 |  | 1.049(0.732,1.501) | 0.795 | 1.080(0.818,1.426) | 0.585 | 1.592(0.939,2.699) | | 0.084 |  | 0.809(0.574,1.142) | | 0.228 | |
| **3. Self-pay** | 0.919(0.563,1.501) | 0.736 | 1.117(0.776,1.609) | 0.551 | 2.143(1.141,4.025) | 0.018 | 0.776(0.447,1.347) | 0.367 |  | 0.999(0.577,1.730) | 0.999 | 1.090(0.740,1.606) | 0.663 | 1.349(0.631,2.884) | | 0.440 |  | 0.588(0.312,1.109) | | 0.101 | |
| **Patient Location** | 0.934(0.901,0.970) | <0.001 | 0.958(0.936,0.980) | <0.001 | 0.894(0.855,0.934) | <0.001 | 0.927(0.895,0.960) | <0.001 |  | 0.949(0.914,0.986) | 0.007 | 0.948(0.924,0.973) | <0.001 | 0.874(0.832,0.917) | | <0.001 |  | 0.927(0.892,0.963) | | <0.001 | |
| **Deyo-Charlson Comorbidity Index** | 1.101(0.974,1.243) | 0.123 | 1.641(1.519,1.772) | <0.001 | 1.094(0.948,1.264) | 0.218 | 1.032(0.922,1.154) | 0.589 |  | 1.030(0.909,1.167) | 0.646 | 1.712(1.574,1.863) | <0.001 | 1.005(0.860,1.174) | | 0.954 |  | 0.977(0.865,1.104) | | 0.714 | |
| **Length of stay at index hospitalization (per 1-day increase)** | 1.007(1.001,1.013) | 0.033 | 1.008(1.005,1.011) | <0.001 | 1.008(1.001,1.016) | 0.032 | 1.006(1.000,1.012) | 0.057 |  | 1.008(1.001,1.014) | 0.020 | 1.006(1.003,1.009) | <0.001 | 1.007(0.999,1.015) | | 0.091 |  | 1.005(0.998,1.011) | | 0.156 | |
| **Severe hospitalization at index hospitalization** | 1.170(1.023,1.339) | 0.022 | 1.410(1.297,1.532) | <0.001 | 1.468(1.259,1.712) | <0.001 | 1.398(1.230,1.588) | <0.001 |  | 0.981(0.850,1.132) | 0.793 | 1.357(1.238,1.487) | <0.001 | 1.182(0.995,1.404) | | 0.058 |  | 1.233(1.072,1.419) | | 0.003 | |

Abbreviations: MHNO, metabolically healthy nonobese; MHO, metabolically healthy obese; MUNO, metabolically unhealthy nonobese; MUO, metabolically unhealthy obese. HR: hazard ratios.

**Supplementary Table 7 Cox proportional hazard analysis evaluating risk of Unplanned Hospitalization in digestive system cancer patients.**

|  | | **30-day Unplanned Hospitalization** | | | | | | |  | **180-day Unplanned Hospitalization** | | | | | | | | | | | |
| --- | --- | --- | --- | --- | --- | --- | --- | --- | --- | --- | --- | --- | --- | --- | --- | --- | --- | --- | --- | --- | --- |
| **Variables** | **HR (95% CI)-neoplasm of upper digestive tract** | **P-value**  **1** | **HR (95% CI)-neoplasm of lower digestive tract** | **P-value**  **2** | **HR (95% CI)-neoplasm of liver** | **P-value 3** | **HR (95% CI)-neoplasm of pancreas** | **P-value**  **4** |  | **HR (95% CI)-neoplasm of upper digestive tract** | **P-value**  **1** | **HR (95% CI)-neoplasm of lower digestive tract** | **P-value**  **2** | | **HR (95% CI)-neoplasm of liver** | **P-value 3** | **HR (95% CI)-neoplasm of pancreas** | | **P-value**  **4** | |  |
| **Metabolic obesity phenotypes** |  |  |  |  |  |  |  |  |  |  |  |  |  | |  |  |  |  | |  | |
| **MHNO** | 1(ref) |  | 1(ref) |  | 1(ref) |  | 1(ref) |  |  | 1(ref) |  | 1(ref) |  | | 1(ref) |  |  | 1(ref) | |  | |
| **MHO** | 1.236(0.965,1.584) | 0.093 | 0.897(0.783,1.028) | 0.119 | 0.836(0.634,1.103) | 0.206 | 1.160(0.894,1.505) | 0.263 |  | 1.073(0.899,1.279) | 0.435 | 0.954(0.891,1.022) | 0.182 | | 1.002(0.854,1.176) | 0.979 |  | 1.004(0.842,1.198) | | 0.962 | |
| **MUNO** | 1.061(0.983,1.146) | 0.130 | 1.075(1.025,1.128) | 0.003 | 1.091(1.011,1.176) | 0.024 | 1.112(1.037,1.192) | 0.003 |  | 1.051(1.002,1.101) | 0.040 | 1.022(0.997,1.048) | 0.086 | | 1.061(1.014,1.111) | 0.011 |  | 1.074(1.028,1.122) | | 0.001 | |
| **MUO** | 1.096(0.965,1.246) | 0.159 | 1.019(0.950,1.094) | 0.595 | 1.154(1.026,1.299) | 0.017 | 1.225(1.098,1.367) | <0.001 |  | 1.020(0.942,1.104) | 0.625 | 0.994(0.957,1.032) | 0.764 | | 1.090(1.010,1.177) | 0.027 |  | 1.124(1.043,1.212) | | 0.002 | |
| **Age (per 1y increase)** | 0.986(0.983,0.989) | <0.001 | 0.990(0.988,0.991) | <0.001 | 0.988(0.985,0.991) | <0.001 | 0.983(0.980,0.985) | <0.001 |  | 0.994(0.992,0.996) | <0.001 | 0.996(0.995,0.997) | <0.001 | | 0.995(0.993,0.997) | <0.001 |  | 0.993(0.991,0.994) | | <0.001 | |
| **Sex (women vs men)** | 0.962(0.897,1.033) | 0.287 | 0.956(0.919,0.995) | 0.026 | 0.992(0.928,1.059) | 0.801 | 0.971(0.920,1.025) | 0.283 |  | 0.992(0.951,1.035) | 0.714 | 0.997(0.977,1.018) | 0.812 | | 1.003(0.963,1.045) | 0.882 |  | 0.995(0.961,1.029) | | 0.756 | |
| **Primary expected payer** |  |  |  |  |  |  |  |  |  |  |  |  |  | |  |  |  |  | |  | |
| **4. No charge/others** | 1(ref) |  | 1(ref) |  | 1(ref) |  | 1(ref) |  |  | 1(ref) |  | 1(ref) |  | | 1(ref) |  |  | 1(ref) | |  | |
| **1. Medicare/Medicaid** | 1.004(0.832,1.211) | 0.966 | 1.243(1.083,1.427) | 0.002 | 1.287(1.066,1.553) | 0.009 | 1.093(0.910,1.315) | 0.342 |  | 1.101(0.978,1.239) | 0.113 | 1.058(0.988,1.133) | 0.109 | | 1.113(0.995,1.246) | 0.062 |  | 1.044(0.926,1.176) | | 0.481 | |
| **2. Private insurance** | 0.811(0.669,0.984) | 0.033 | 0.974(0.847,1.121) | 0.717 | 1.170(0.962,1.422) | 0.116 | 0.999(0.828,1.205) | 0.992 |  | 1.004(0.888,1.134) | 0.955 | 0.980(0.914,1.051) | 0.572 | 1.045(0.929,1.175) | | 0.467 |  | 0.981(0.868,1.108) | | 0.755 | |
| **3. Self-pay** | 0.801(0.588,1.091) | 0.160 | 0.977(0.800,1.192) | 0.817 | 0.971(0.711,1.326) | 0.855 | 1.025(0.771,1.363) | 0.864 |  | 0.991(0.820,1.198) | 0.926 | 0.988(0.891,1.094) | 0.810 | 0.974(0.811,1.170) | | 0.778 |  | 1.070(0.884,1.296) | | 0.487 | |
| **Patient Location** | 0.940(0.920,0.961) | <0.001 | 0.972(0.959,0.984) | <0.001 | 0.952(0.932,0.973) | <0.001 | 0.939(0.922,0.957) | <0.001 |  | 0.976(0.964,0.989) | <0.001 | 0.990(0.984,0.997) | 0.006 | 0.973(0.961,0.986) | | <0.001 |  | 0.973(0.962,0.984) | | <0.001 | |
| **Deyo-Charlson Comorbidity Index** | 1.277(1.187,1.374) | <0.001 | 1.636(1.567,1.708) | <0.001 | 1.241(1.154,1.336) | <0.001 | 1.160(1.092,1.233) | <0.001 |  | 1.065(1.020,1.113) | 0.005 | 1.150(1.124,1.177) | <0.001 | 1.039(0.994,1.087) | | 0.089 |  | 1.002(0.964,1.041) | | 0.937 | |
| **Length of stay at index hospitalization (per 1-day increase)** | 0.999(0.995,1.004) | 0.823 | 1.002(0.999,1.005) | 0.114 | 1.002(0.996,1.007) | 0.521 | 0.995(0.989,1.001) | 0.094 |  | 1.000(0.997,1.003) | 0.858 | 1.000(0.998,1.001) | 0.828 | 1.001(0.997,1.004) | | 0.644 |  | 0.999(0.996,1.002) | | 0.438 | |
| **Severe hospitalization at index hospitalization** | 1.000(0.918,1.091) | 0.993 | 1.228(1.166,1.294) | <0.001 | 1.054(0.962,1.154) | 0.263 | 1.077(0.987,1.174) | 0.095 |  | 0.954(0.905,1.006) | 0.083 | 1.051(1.020,1.083) | 0.001 | 0.946(0.893,1.002) | | 0.060 |  | 0.998(0.948,1.051) | | 0.948 | |

Abbreviations: MHNO, metabolically healthy nonobese; MHO, metabolically healthy obese; MUNO, metabolically unhealthy nonobese; MUO, metabolically unhealthy obese. HR: hazard ratios
